# Principles and Practice of Surgery

**Published:** 1878-10

**Authors:** 


### BIBLIOGRAPHICAL.

Principles and Practice of Surgery, being a Treatise on Surgical Diseases and Injuries. By D. Hayes Agnew, M.D., LL.D., Professor of Surgery in the Medical Department of the University of Pennsylvansa. Profusely illustrated, in two volumes. Philadelphia: J. B. Lippincott & Co. London: 16 Southampton street, Covent Garden. 1878.

The first volume of this work has just been received from the publishers at Philadelphia, and, we suppose, will be followed by the second volume so soon as it is issued from the press.

The volume before us contains about 1,040 pages, and, from the cursory examination we have been able to give it, appears to be so arranged and illustrated as to give the greatest advantages in the study and practice of the science and art of surgery.

We shall take pleasure in giving a more extended notice of the work when both volumes are published.

